# Evidence of osteoarthritis disease modification with a Sn-117m microparticle device: a review and validation in mammalian models

**DOI:** 10.3389/fvets.2025.1621296

**Published:** 2025-07-11

**Authors:** Alison Bendele, Cynthia A. Doerr, Gilbert R. Gonzales, Robert Menardi, Eric Schreiber, Nigel R. Stevenson

**Affiliations:** ^1^Inotiv, Inc., Boulder, CO, United States; ^2^Serene, LLC, The Woodlands, TX, United States; ^3^Exubrion Therapeutics, Inc., Gainesville, GA, United States

**Keywords:** degenerative joint disease, pain management, radiosynoviorthesis, Sn-117m, synovitis, disease modifying osteoarthritis drugs, veterinary medicine, DMOAD

## Abstract

Osteoarthritis (OA) is a progressive joint disorder affecting mammals as well as many non-mammalian animals, characterized by cartilage degradation and synovial inflammation, yet current treatments focus solely on symptom relief rather than disease modification. Radiosynoviorthesis (RSO) using commercially available homogeneous Sn-117m microparticles (HTM) (Synovetin OA®, Exubrion Therapeutics, Inc.) injected into the arthritic joint space targets synovitis—a critical driver of OA progression—offering a novel therapeutic approach. This review collates preclinical and clinical evidence demonstrating HTM’s potential to alter OA’s natural course in mammals. We explore OA pathogenesis, emphasizing synovitis, macrophage activity, and the inflammatory cycle, alongside RSO’s historical use and Sn-117m’s mechanism: low-energy electrons targeting the inflamed synovium. Preclinical studies in male Lewis rats with meniscal tear-induced OA revealed reductions in synovial inflammation, cartilage damage, and osteophyte formation, suggesting a disease-modifying effect. Clinical trials in dogs with elbow OA further substantiate these findings: a Grade 1 & 2 OA study showed durable lameness improvement over 12 months—long after Sn-117m’s 13.9-day half-life (t_½_)—indicating benefits beyond the active irradiation period. A reinjection study found that 50% of dogs exhibited no OA progression on imaging, suggesting HTM’s capacity to slow disease advancement. Unlike NSAIDs, which relieve pain without addressing etiology, Sn-117m targets the source of inflammation. Corticosteroids are effective anti-inflammatories when delivered into the joint, but can cause thinning of cartilage, bone loss, and joint instability. This review substantiates Sn-117m RSO as a transformative veterinary therapy, bridging preclinical insights with clinical outcomes that strongly supports a positive disease-modifying mechanism.

## Introduction

Osteoarthritis (OA) stands as one of the most prevalent chronic conditions impacting mammals, affecting over 20% of adult dogs, with elbow joints particularly susceptible due to biomechanical stress and breed-specific predispositions, such as those observed in Labrador Retrievers, German Shepherds, and Rottweilers (1). This degenerative joint disease manifests through cartilage degradation, subchondral bone remodeling, osteophyte formation, and synovial inflammation, collectively leading to debilitating pain, lameness, and reduced quality of life. In veterinary medicine, OA poses significant welfare challenges and economic burdens, as pet owners seek interventions to alleviate suffering and preserve mobility. Current therapeutic options—nonsteroidal anti-inflammatory drugs (NSAIDs) like carprofen or meloxicam, monoclonal antibodies such as bedinvetmab, analgesics such as tramadol or gabapentin, intra-articular corticosteroids, and supportive measures like physical therapy or weight management—primarily palliate symptoms. These treatments reduce discomfort and enhance function short-term but fail to slow disease progression, highlighting the need for disease-modifying OA drugs (DMOADs) or devices (DMOADevices) targeting structural and inflammatory etiologies (2).

Historically, OA was viewed as a mechanical wear-and-tear disorder from repetitive joint loading, trauma, or congenital abnormalities. However, recent research elevates synovitis as a central driver of OA’s initiation and progression, detectable even in early stages (3, 4). Synovitis amplifies cartilage breakdown via pro-inflammatory mediators, sensitizes nociceptors, and sustains joint destruction through immune activation. Macrophages, key immune cells in the synovium, exacerbate this process by releasing cytokines like IL-1β and TNF-α, driving inflammation and ultimately cartilage degradation (5). This shift redirects therapeutic efforts toward mitigating synovial inflammation, including macrophage activity, to alter disease trajectory.

Radiosynoviorthesis (RSO) emerges as a compelling option. Introduced in the 1950s for human arthropathies like rheumatoid arthritis (6), RSO used intra-articular radioactive particles to ablate hyperplastic synovium. However, Sn-117m, a novel isotope in homogeneous microparticles (HTM) form, refines RSO for veterinary OA. Its low-energy electrons target synovial tissue through non-ablative mechanisms, spares cartilage and bone, and possesses a 13.9-day t_½_ ensuring controlled exposure. Recent trials in dogs with elbow OA demonstrate Sn-117m’s ability as a device to alleviate symptoms and modify structural progression (7).

This review evaluates Sn-117m’s potential as a disease-modifying OA Device (DMOADevice), emphasizing its relevance to veterinary medicine. We examine OA pathogenesis, focusing on synovitis, macrophage activity, and the inflammatory cycle driving joint destruction. We trace RSO’s evolution from human medicine to veterinary applications and highlight Sn-117m’s mechanism, emphasizing its suitability for OA therapy. Preclinical studies using rat models show HTM’s therapeutic disease modifying properties. Clinical trials in dogs with elbow OA also support these findings —including studies in dogs with mild/moderate OA (“Grade 1 and 2”) demonstrating sustained function, similar findings in dogs with severe radiographic OA (“Grade 3”), as well as reinjection studies measuring radiographic disease progression. By targeting OA’s inflammatory etiology, Sn-117m shows promise to transform veterinary care, filling a gap in disease-modifying treatments and enhancing outcomes for companion animals with this prevalent condition.

## Pathogenesis of osteoarthritis

Osteoarthritis arises from a multifaceted interplay of biomechanical and biochemical factors that progressively undermine joint homeostasis, ultimately leading to its deterioration. Mechanical stress—stemming from acute trauma, conformational abnormalities, chronic overloading due to obesity, or repetitive motion typical in active canine breeds—initiates microdamage within articular cartilage, compromising its capacity to absorb and distribute mechanical loads effectively. This initial insult disrupts the extracellular matrix (ECM), a complex network of collagen II and proteoglycans that provides cartilage with its structural integrity. In response, chondrocytes—the resident cells of cartilage—release matrix fragments, including fibronectin and aggrecan breakdown products, which function as damage-associated molecular patterns (DAMPs). These DAMPs ignite an inflammatory cascade that marks the onset of OA’s pathological progression (8).

Inflammation escalates cartilage destruction with activated chondrocytes and synoviocytes producing a suite of pro-inflammatory cytokines which upregulate the expression of matrix metalloproteinases and aggrecanases (ADAMTS-4 and -5). These enzymes degrade collagen and proteoglycans, eroding the ECM and disrupting cartilage integrity. Concurrently, subchondral bone undergoes significant pathological remodeling: osteoclasts increase bone resorption, leading to sclerosis and abnormal thickening, while osteoblasts deposit excess bone at the joint margins forming osteophytes, the bony outgrowths that exacerbate joint instability, impinging on surrounding soft tissues.

Synovial inflammation emerges as a pivotal amplifier in this degenerative spiral. Activated synoviocytes secrete additional inflammatory mediators which promote angiogenesis within the synovium and contribute to synovial effusion, manifesting as joint swelling and discomfort. In canine models, such as those with surgically destabilized stifle joints, histopathological analyses consistently reveal synovial thickening and cartilage erosion as early events. This suggests that inflammation precedes, drives and perpetuates OA by chronic mechanical stress (9). Pain, a hallmark symptom of OA, arises from both the mechanical irritation of damaged joint tissues and the inflammatory sensitization of nociceptors, mediated by cytokines and other signaling molecules.

The chronicity of OA’s pathogenesis distinguishes it from acute joint injuries, as the disease evolves into a self-sustaining cycle of destruction. Cartilage loss destabilizes the joint, increasing mechanical stress on remaining tissues, which in turn amplifies inflammation and bone remodeling. This triad of cartilage breakdown, subchondral bone alteration, and synovial-driven inflammation defines OA’s progressive nature.

Synovitis, defined as inflammation of the synovial membrane, has transitioned from a perceived secondary consequence of OA to a widely acknowledged driver of its initiation and progression, often preceding significant cartilage loss. Synovitis is now routinely detected in early OA through imaging and histological examination (10). In canine elbow OA synovial hyperplasia and effusion are frequent observations, exhibiting a strong correlation with disease severity, lameness scores, and owner-reported pain levels, thus underscoring its clinical significance in veterinary patients.

Within the inflamed synovium an active immunological environment takes shape, characterized by infiltration of macrophages, lymphocytes, and neutrophils. Synovial fluid analyses from affected canine joints reveal markedly elevated concentrations of pro-inflammatory mediators, indicative of a robust and sustained inflammatory state. Histologically, OA-affected synovium may display distinctive features including villous hypertrophy, increased cellularity, and fibrous deposition which signal chronic activation and structural remodeling. Synoviocytes directly contribute to cartilage degradation by secreting proteolytic enzymes that erode the ECM and compromise cartilage integrity. Additionally, they foster an inflammatory microenvironment that recruits additional immune cells, amplifying joint destruction through a cascade of cellular and molecular interactions.

Synovitis and inflammatory mediators such as PGE2 and nerve growth factor (NGF), sensitize nociceptors and heighten pain perception. In preclinical models such as the rat meniscal tear model, synovial inflammation manifests within days of surgical intervention and early joint injury, preceding the development of osteophytes and cartilage destruction, which strongly suggests a causal role in driving disease advancement. In veterinary practice, synovitis is a consistent underlying condition across OA-affected joints, with particular prominence in weight-bearing sites like the elbow and stifle, where it exacerbates biomechanical stress and accelerates joint deterioration.

The recognition of synovitis as a primary driver shifts the therapeutic paradigm toward strategies that directly mitigate inflammation at its synovial source. By addressing this early and sustained inflammatory component, interventions can potentially interrupt the progression of OA, offering a more effective means of altering its course compared to therapies that merely mask downstream effects like pain or stiffness. Effective therapies must therefore address three components—mechanical integrity, inflammatory activity, and structural stability—to meaningfully alter the disease’s trajectory. Current palliative approaches fall short of this goal, highlighting the need for innovative strategies, such as those targeting synovial inflammation, to break the cycle and offer genuine disease modification in OA-affected mammals.

## The role of macrophages

Macrophages serve as central orchestrators of the inflammatory pathology underpinning OA, exerting a profound influence on disease progression. Within the synovial environment, resident macrophages, supplemented by infiltrating monocytes, undergo polarization into a pro-inflammatory M1 phenotype in response to several immune chemical messengers. This shift redirects their function from tissue repair toward destructive activity (5).

In canine OA, synovial biopsies from elbow joints reveal dense macrophage infiltration, with their presence becoming more pronounced as the disease advances from mild to severe stages. Cartilage debris, generated by joint wear, activates macrophages, triggering the innate immune system and amplifying synovial hyperplasia. This feedback loop sustains the “vicious inflammatory cycle,” driving chondrocyte apoptosis and ECM degradation through the action of matrix metalloproteinases (MMPs) and aggrecanases. Beyond structural damage, macrophages contribute directly to pain, secreting PGE2 and NGFs to sensitize peripheral nociceptors, thereby exacerbating clinical symptoms.

In preclinical rodent OA models where macrophages are depleted, both synovial inflammation and cartilage loss are significantly reduced, indicating that these cells are not mere responders but active propagators of disease pathology. In companion animal OA, the density of macrophages within the synovium correlates closely with radiographic markers of progression, such as osteophyte growth and cartilage thinning, linking their inflammatory activity to structural deterioration.

Sn-117m RSO capitalizes on this mechanism by causing macrophage ablation thereby diminishing the inflammatory output and potentially modifying disease progression. This targeted intervention highlights macrophages as both a driver of OA pathology and a viable therapeutic target in the quest for effective disease management.

Synovial macrophages amplify this vicious cycle by secreting immune chemical messengers and activating the innate immune system, thus promoting synovial angiogenesis, effusion, and thickening, exacerbating joint swelling and mechanical dysfunction. In canine OA this inflammatory feedback loop aligns with radiographic evidence of advancing pathology, such as osteophyte formation and subchondral bone sclerosis, as inflamed synovium contributes to both bony remodeling and cartilage loss.

The chronic nature of this cycle sets OA apart from transient joint injuries. Each round of cartilage degradation and inflammation intensifies subsequent iterations, locking the joint into a degenerative spiral that resists spontaneous resolution. Pain emerges as a dual consequence: mechanical stress from damaged tissues and inflammatory sensitization of nociceptors by mediators like PGE2, linking the cycle directly to clinical decline. This relentless progression underscores the challenge of managing OA with palliative measures alone, as the underlying inflammatory engine continues unabated.

Breaking this feedback loop demands therapies that suppress synovial inflammation and macrophage activity while preserving joint integrity. Sn-117m RSO fulfills this role by targeting the inflamed synovium, ablating its inflammatory components, and potentially halting OA’s advancement.

## History and use of radiosynoviorthesis

Radiosynoviorthesis, also known as radiosynovectomy, emerged in the 1950s as an innovative treatment for refractory synovitis in human rheumatoid arthritis, pioneered by clinicians seeking alternatives to surgical synovectomy (11). Early practitioners employed beta-emitting isotopes, such as Gold-198, Phosphorous-32 and Yttrium-90 (Y-90) delivered via intra-articular injection to target hyperplastic synovium with localized radiation. This approach effectively reduced synovial mass and inflammation, alleviating pain and effusion in 60–80% of patients, thereby establishing RSO as a viable therapeutic option (12). By the 1970s, advancements introduced isotopes like Rhenium-186 (Re-186) and Erbium-169 (Er-169), refining the technique by tailoring the profile of radiation range in tissue to joint size—larger joints like knees benefited from Y-90, mid-size joints like ankles benefited from Re-186, while smaller joints like fingers utilized Er-169.

The mechanism of RSO hinges on the phagocytosis of radioactive microparticles by synovial macrophages and synoviocytes, followed by radiation-induced apoptosis of these and other inflammatory cells. This targeted ablation diminishes synovial hyperplasia without ablative effects to adjacent cells, and curbs the production of pro-inflammatory mediators, offering a localized anti-inflammatory effect with minimal systemic exposure. Beta emitters like Y-90, with penetration depths ranging from 3 to 11 mm, excel in treating large joints but pose a risk of collateral damage to cartilage and bone in smaller joints due to their broader energy distribution. In contrast, Er-169, with a penetration range of 0.3–1 mm, was developed to address this limitation, emphasizing the importance of isotope specificity based on joint anatomy (13).

In human OA, RSO gained traction as a means to manage inflammation-driven refractory synovitis and chronic hemophilic arthropathy and pain, particularly in cases resistant to conventional therapies. While its application in OA remains less extensive than in rheumatoid arthritis, it has become a standard adjunctive treatment particularly in regions of Europe and Asia, where clinical experience has demonstrated its effectiveness and safety in the treatment of OA (6, 12, 13). Veterinary applications, however, have lagged, with only anecdotal reports of Y-90 and Ho-166 (14) use in equine joints to address synovitis, reflecting a slower adoption in animal medicine despite the parallels in joint pathology across species.

HTM represents a significant evolution in RSO, adapting this established technique for companion animals like dogs. Building on decades of human clinical experience, Sn-117m offers a refined approach tailored to veterinary needs for synovial joints. Its introduction addresses OA’s inflammatory component with a precision and safety profile optimized for targeted synovial therapy, minimizing risks to surrounding tissues. This advancement positions RSO as a bridge between human and veterinary medicine, leveraging historical insights to tackle a prevalent condition in companion animals with a fresh, isotope-specific strategy.

RSO, utilizing the unique radioisotope Sn-117m in its HTM formulation, is now accessible to veterinarians in companion animal practice. Practices that choose to offer this therapy are required to obtain a Radioactive Materials License (RML). An RML is an official authorization from the Nuclear Regulatory Commission (NRC) or state regulatory agency, to handle radioactive materials. The RML allows practices to possess, use, store, and handle Sn-117m for the purpose of medical treatments. Once training, equipment, and facility requirements are met, the veterinarian is authorized to use HTM.

## Sn-117m: mechanism of action

Sn-117m (tin-117 m) is a metastable isotope with ideal characteristics for the use of RSO in OA (15), offering a therapeutic mechanism to address synovial inflammation. It emits low-energy conversion electrons (~140 keV) with a tightly constrained penetration range of <0.3 mm, delivering radiation that leads to apoptosis of the phagocytosing cells (macrophages and synoviocytes in the synovial lining) while sparing deeper structures such as articular cartilage, subchondral bone, and periarticular tissues. This shorter range of emission distinguishes Sn-117m from beta-emitting isotopes like Y-90, which penetrate 3–11 mm and risks unintended damage in smaller joints. Sn-117m’s 13.9-day t_½_ provides a therapeutic window sufficient to achieve inflamed synovial treatment after which it decays into stable Sn-117 via gamma emission (159 keV), detectable for dosimetric monitoring and subsequently biologically inert.

Formulated as HTM, Sn-117m is designed to remain suspended within the joint space following intra-articular injection, resisting leakage into systemic circulation—a marked improvement over earlier microparticles that occasionally dispersed via lymphatic pathways. Once administered, synovial macrophages and synoviocytes engulf the microparticles, a process that positions the isotope within the source of inflammation. The emitted conversion electrons induce DNA single and double strand breaks within these cells, triggering apoptosis and reducing the population of inflammatory cells. This targeted ablation curtails the production of pro-inflammatory cytokines—interleukin-1β (IL-1β), tumor necrosis factor-α (TNF-α), and interleukin-6 (IL-6)—and diminishes synovial hyperplasia, directly counteracting synovial inflammation fueling OA progression.

In the context of OA treatment, Sn-117m’s mechanism aligns with the disease’s pathology, particularly its reliance on synovial inflammation as a central propagator. By treating the inflamed synovium, Sn-117m interrupts the vicious inflammatory cycle, reducing macrophage-driven cytokine release and preserving cartilage integrity by alleviating the enzymatic assault from MMPs and aggrecanases. Unlike systemic anti-inflammatory drugs, which broadly suppress inflammation with off-target effects, Sn-117m’s localized action minimizes systemic toxicity, offering a focused therapeutic impact. Furthermore, clinical evidence from canine trials suggests that its effects persist well beyond its radioactive lifespan, indicating a lasting modification of joint homeostasis rather than a mere transient reduction in symptoms (7, 16, 17).

This durability underscores Sn-117m’s potential as a DMOADevice, distinguishing it from palliative therapies that address only pain or stiffness. Its specific joint treatment and safety profile make it particularly well-suited for veterinary applications, where joint sizes—such as the canine elbow—demand interventions that avoid collateral damage while accommodating patient tolerability. Compared to broader-spectrum radiation therapies used historically in RSO, Sn-117m represents a lower energy, minimally invasive alternative, offering a targeted approach to OA management that leverages its unique radiophysical properties to achieve both symptomatic relief and possible structural preservation (see section on Rat Model Studies below).

## Current treatment options

The management of OA in veterinary medicine currently relies on a multimodal toolkit (the discussion below is not meant to be comprehensive review of current treatment options as these already exist, e.g. (19)). Multiple options, often employed in combination, primarily target the pain that is a defining feature of this progressive disease. They are not generally intended to modify the course of the underlying inflammatory disease or address progression.

NSAIDs, such as carprofen and meloxicam, dominate first-line therapy by inhibiting cyclooxygenase enzymes, thereby reducing prostaglandin-mediated pain and inflammation. The emphasis has always been on analgesia as opposed to reducing inflammation. These drugs improve mobility in dogs with elbow OA, offering significant symptomatic relief. Prolonged administration carries risks of gastrointestinal ulceration, renal toxicity, and hepatic stress, necessitating appropriate health monitoring with long-term use. A study in human OA revealed no long-term benefit of NSAIDs for the progression of OA, and suggested that they may accelerate radiographic signs of progression (20).

Nutraceuticals, such as glucosamine and chondroitin sulfate, enjoy widespread use among pet owners hopeful for cartilage support (21). Randomized controlled trials, however, reveal variable effectiveness, with inconsistent reductions in lameness or demonstrable slowing of cartilage loss, casting doubt on their reliability as standalone therapies. Polysulfated glucosaminoglycan injections (8 injections over a 4-week period) are also used to treat canine arthritis. Despite their popularity the mechanism of action is not known and the benefits when they occur diminish over time. At this time, a single polysulfated glycosaminoglycan product (Adequan, American Regent Animal Health) is the only FDA approved treatment designated as a disease-modifying osteoarthritis drug (DMOAD).

A host of intra-articular injectable therapies are also employed. Intra-articular corticosteroids, such as triamcinolone or methylprednisolone, provide rapid anti-inflammatory effects by suppressing cytokine production, reducing synovial effusion and pain. Yet, repeated injections accelerate cartilage catabolism and weaken joint structures, paradoxically exacerbating degeneration over time (22).

Hyaluronic acid injections are employed to restore synovial fluid viscosity and enhance joint lubrication, aiming to alleviate friction-related discomfort. Despite their popularity, clinical evidence supporting structural benefits remains inconsistent, with benefits often transient and limited to mild cases. Other intra-articular modalities have gained in popularity, such as platelet-rich plasma (PRP) and stem cells, which are intended to promote angiogenesis and chondrocyte proliferation.

Surgical interventions offer more definitive mechanical correction for conformational abnormalities or removal of osteochondral fragments. Surgical treatment of the osteoarthritic joint ranges from arthroscopy to remove loose debris and smooth cartilage surfaces, to total joint replacement to address end-stage OA. These options can be invasive, expensive, and are often reserved for severe cases unresponsive to medical management, making them inaccessible or impractical for many patients.

Physical therapy and weight management serve as valuable adjuncts, strengthening periarticular muscles and reducing joint load, respectively. While these measures enhance mobility and mitigate mechanical stress, they fail to address the inflammatory pathology at OA’s core, leaving the disease’s progression unchecked.

Collectively, these treatments excel at mitigating OA symptoms—pain, stiffness, and lameness—which is critical to providing temporary relief from the pain of OA that improves quality of life in the short term. However, they fall short of targeting synovitis or halting cartilage degradation, the etiological drivers that perpetuate OA’s advance. This palliative focus highlights a critical gap in veterinary care: the absence of therapies capable of modifying the disease’s course at its inflammatory root. Sn-117m RSO emerges as a promising candidate to fill this void, offering a targeted approach that addresses synovial inflammation directly, potentially altering OA’s trajectory while current options mitigate its downstream consequences.

## Preclinical evidence: rat model studies

A surgically induced medial meniscal tear (MMT) OA model was established in Lewis rats as previously described (23). The rats were randomized into the groups shown in Table 1 and injections occurred in the right knee except for Group 4 (no surgery) which received injections in both knees for safety evaluation (24).

**Table 1:** Rat OA trial design.

Rat OA GLP Study							
Date of procedure/sacrifice		−1 wk	0 wk	1 wk	4 wk	6 wk	10 wk
Group	# animals						
Group 1 (Model Control)	15	Surgery	None	4	4	4	3
Group 2--2uCi	31	Surgery	Sn-117m microparticles	10	10	11	
Group 3--10uCi	36	Surgery	Sn-117m microparticles	11	11	11	3
Group 4--10uCi (no disease)	8	No Surgery	Sn-117m microparticles	2	2	2	2
Total OA study	90						

OA was surgically induced in study rats via MMT surgery 7 days prior to a single intra-articular injection of 2 μCi (low dose) or 10 μCi (high dose) HTM. The dosing is done 1 week post-surgery to allow the synovium and joint capsule sufficient time to heal so that the injected material will be retained within the space and not leak out. Two control groups were included: Group 1-MMT induced OA had no microparticle injections; Group 4-had no surgery but received Sn-117m microparticle injections (each knee received either high or low dose). Data collection and analysis completed at various time-points included blood work, urine and fecal radiation excretion, histopathology, and bio-distribution. Rats were sacrificed at 7 days, 28 days (2 t_½_), 42 days (3 t_½_), and 70 days (5 t_½_) after surgery. The animals listed on the table below were divided into cohorts for each timepoint. Model controls had 3-4/group at each time point and 2 or 10 μCi treated groups had 10 for the 1-, 4-, and 6-week terminations and 2 or 3 for the 10-week endpoint.

All animals maintained normal physical activity throughout the study. Clinical evidence of pain as evidenced by limping is rare in this model so analgesics are only given if animals show evidence of pain. In animals with full biodistribution, excluding obvious missed injections, joint retention of the Sn-117m micro particles was 99.7%. Urinary and fecal excretion of the isotope was typically at <2 X background by 24 h. Urinary and fecal cumulative radiation excretion averaged <0.005% of the administered dose for all 4 days measured.

**Figure 1:**
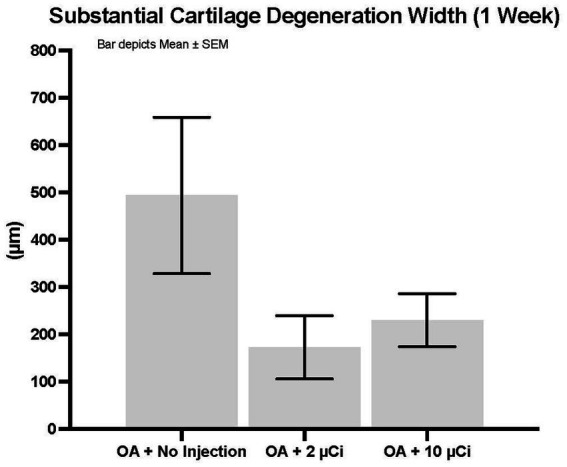
Substantial cartilage degeneration width. Treatment with Low or high dose Sn-117m microparticles resulted in 65% or 53% inhibition, respectively of this parameter at 1 week. Substantial cartilage degeneration width is a micrometer measure of the width of cartilage degeneration across the medial tibial plateau (area at risk for greatest pathology in this model) that extends through 50% of the thickness of the cartilage. Basically, it excludes minimal or mild cartilage degeneration and focuses on the more severe pathology.

**Figure 2:**
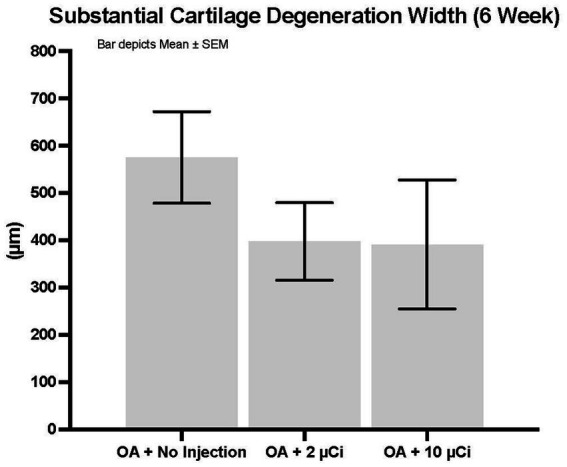
Substantial cartilage degeneration width. Treatment with Low or high dose Sn-117m microparticles resulted in 31 or 32% inhibition, respectively of this parameter at 6 weeks.

**Figure 3:**
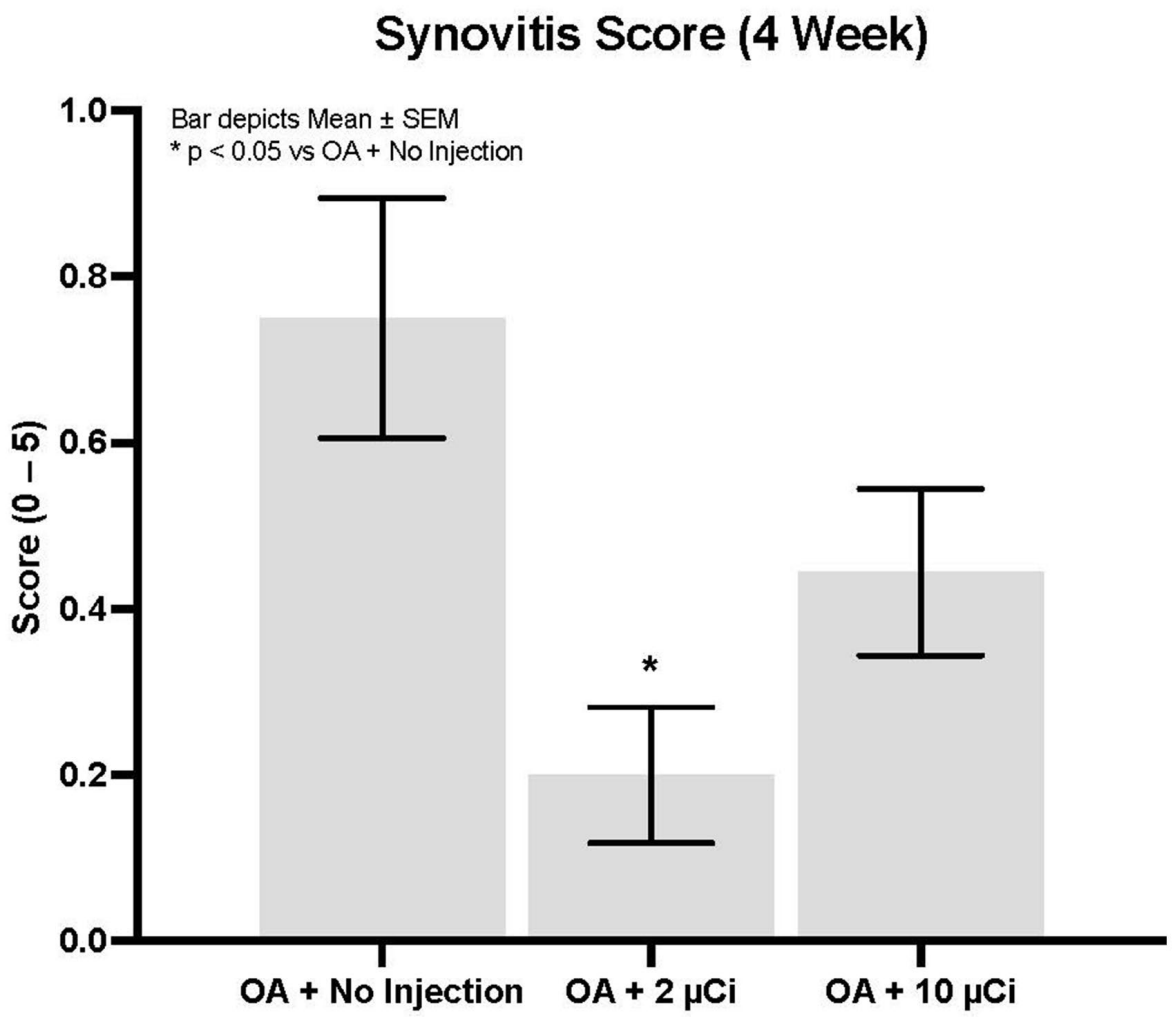
Synovitis. Low dose Sn-117m micro particles show significantly less synovitis (71% inhibition) than the OA model control at 4 weeks. The high dose inhibited synovitis by 41%.

Histopathology revealed that both treatment groups had reduced medial tibial lesion severity compared to controls, with mild beneficial effects on some parameters noted at 7, 28, and 42 days (Figures 1–3). This positive effect was not sustained for 70 days (which is not unexpected in this rapidly progressive rat model). In all figures an “*” identifies statistical significance compared to the model control without treatment (first column).

These results demonstrate that this unique Sn-117m microparticle product, when accurately delivered intraarticularly, is nearly completely retained, thus mitigating the likelihood of unintended distal tissue irradiation. Following administration, there are no clinical safety concerns as noted by normal rat behavior and microscopic evaluation of joints from rats that received Sn-117m treatments but no MMT surgery. Fecal and urinary radiation excretion is nominal immediately following Sn-117m micro particle administration, suggesting the ability to rapidly release rats from radiation isolation. Histopathology indicates disease modifying positive therapeutic effect of both doses.

## Clinical evidence: canine elbow OA trials

Clinical trials in dogs with elbow OA provide compelling translational validation of HTM’s preclinical promise. Four distinct studies were performed to evaluate effectiveness and safety of HTM using multiple assessment methods over a 1-year post-treatment period. The first trial demonstrated safety in five normal dogs (25). The second trial evaluated treatment response in dogs with mild to moderate (Grade 1 and 2) elbow OA (7). A third trial evaluated dogs with severe (Grade 3) elbow OA (16), and a fourth trial evaluated dogs with Grade 1and/or 2 OA that were reinjected with HTM at least 1 year following initial treatment (17).

**Figure 4:**
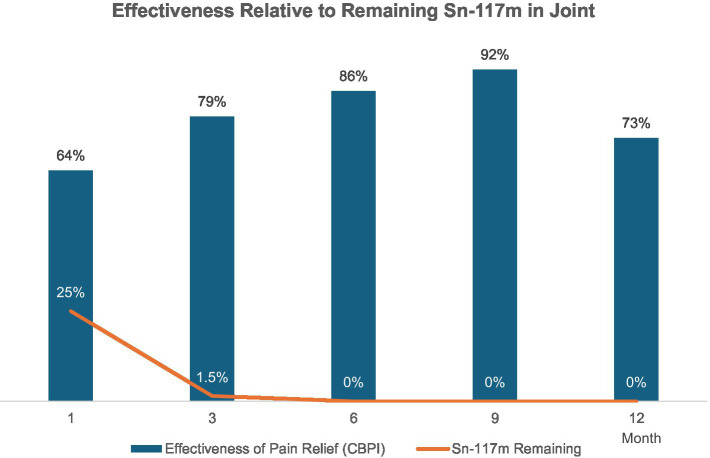
Effectiveness rate relative to remaining Sn-117m in joint (CBPI scores in Grade 1 and 2 Elbow OA Study). Improvement in the majority of dogs was noted at 1 month post-injection, and the percentage of dogs exhibiting improvement continued to increase through 9 months. Clinical effect was apparent long after complete decay of the radiotherapeutic agent.

**Figure 5:**
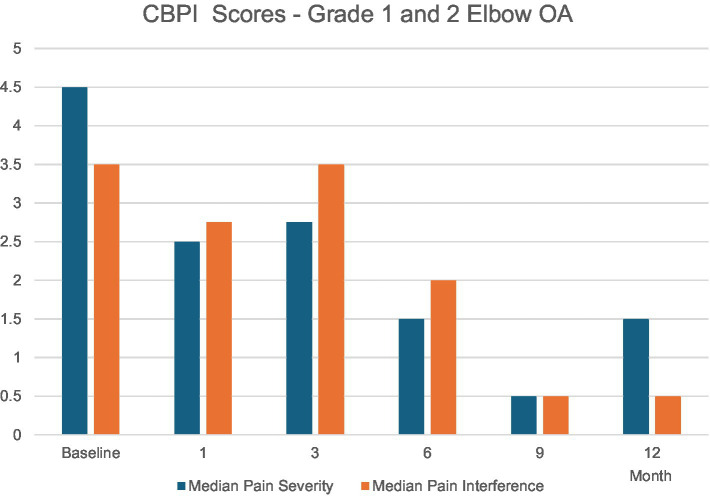
Median owner-reported improvement across the group of treated dogs demonstrating a pattern of progressive improvement through 9 months in both severity and functional impact. Both scores were statistically significant over baseline at all time points (except at 3 months where only the Median Pain Severity score was statistically significant).

The Grade 1 and 2 study included dogs with mild-to-moderate elbow OA as identified by clinical signs of pain or lameness localized to the elbow joint, and radiographic evidence of minimal osteophytes and sclerosis. These conditions represent the disease’s earlier stage where intervention might yield maximal benefit. Dogs taking systemic analgesics prior to the study period were included as long as elbow pain and lameness were persistent in the face of analgesic use. Owners were encouraged to decrease or eliminate analgesic usage upon regression of clinical signs of lameness. Dogs were randomly placed in a low, medium, or high dose group and received a single intraarticular dose within those groups based on individual body surface area. Effectiveness compared to pretreatment baseline condition was assessed over 12 months post-treatment using multiple metrics at prescribed post-treatment intervals. A validated survey tool and scoring system, the Canine Brief Pain Inventory (CBPI), was used for dog owners to quantify their pet’s pain severity and functional impact as well as quality of life. Assessment and scoring of lameness by a veterinary clinician was also employed. Force plate gait analysis measuring peak vertical force as an objective indicator of weight-bearing capacity was used in a subset of patients. Treatment effectiveness was determined by CBPI scores and corroborated by statistical agreement with clinician-assessed lameness scores and force plate gait analysis. By these criteria, 75% of dogs experienced significant improvement (7). In the medium-dose group (the dose ultimately chosen for commercialization) 92% of the dogs improved. Improvements in mean pain severity scores and/or pain interference scores were significant at all time intervals assessed through 12 months. Remarkably, the scale of these improvements increased steadily up to 9-months post-treatment and continued to persist to the 12-month mark—fully 9 months after Sn-117m’s virtually complete radioactive decay. In the medium-dose group, 92% of the dogs were improved compared to baseline at 9 months, with 73% still showing improvement at 12 months. Mean Pain Severity scores in this group at 9 months were improved by 89% (7). This sustained effectiveness extending well beyond the isotope’s active period, strongly suggests a structural disease-modifying effect rather than a transient anti-inflammatory response (see Figure 4). Furthermore, similar results (see Figure 5) were apparent when evaluating mean and median owner-reported improvement across the group of treated dogs (26). The same pattern of progressive improvement through 9 months in both severity and functional impact was apparent. The likely mechanism involves eliminating synovial inflammatory cells, reducing chronic inflammation and resetting joint homeostasis, preserving cartilage integrity over an extended time.

The Grade 3 study targeted dogs with severe elbow OA, characterized by extensive osteophytes and significant loss of structure, representing a more challenging therapeutic context. These dogs received the same HTM dosing as the mid-group in the previous study, and outcomes were tracked similarly over 12 months. The overall success rate of this study was 71%, and pain severity and interference scores were improved at all time points through 12 months. Once again, the degree of improvement increased through 9 months and persisted through 12 months. At the 9-month assessment, mean Pain Severity scores had improved by 60% from baseline, and mean Pain Interference scores had improved by 66% (16). While less pronounced than in the Grade 1 and 2 study, these results indicate HTM’s effectiveness extends across OA severity levels, likely by reducing synovial inflammation even in joints with advanced structural damage.

The reinjection study included a subset of dogs from the Grade 1 and 2 study that received a second HTM injection at least a year following the first, a scenario testing HTM’s safety and sustained impact with repeat injections. These dogs received the second HTM dose at the same mid-dose range of the previous studies and a new baseline was established for pain and lameness prior to the second injection. At 6 months post-reinjection, 67% of subjects were improved from the newly established baseline, and severity and interference scores were reduced through 6 months post-reinjection (17).

Notably, approximately half of the elbows that had been injected twice did not show signs of disease progression based on radiographs, CT, and MRI performed over the 18 + months of the combined studies (17). While radiographic signs of OA might be expected to worsen over time, no conclusion can be drawn from this study alone. A future area of investigation might compare disease progression in treated vs. untreated dogs over an extended period of time.

Together, the durable response beyond radioactive decay and the reinjection study’s 50% non-progression rate—highlight HTM’s potential to alleviate pain, improve function and delay OA advancement, distinguishing it as a potential DMOADevice in veterinary medicine.

## Discussion

HTM directly targets the inflammatory drivers of OA, setting it apart from conventional palliative therapies which primarily focus on symptom management. Preclinical studies using male Lewis rats demonstrated HTM’s effectiveness in a meniscal tear model, revealing reductions in synovial inflammation, cartilage damage, and osteophyte formation—outcomes consistent with Sn-117m’s mechanism of suppressing pro-inflammatory cytokines like IL-6 and TNF-α. These findings translate robustly into clinical trials in dogs with elbow OA, where the Grade 1and 2 study showcased a 12-month durability of lameness improvement, persisting 9 months beyond Sn-117m’s radioactive decay (13.9-day t_½_). This sustained benefit suggests a lasting alteration of joint homeostasis, preserving cartilage integrity and potentially delaying degenerative progression—a hallmark of a DMOADevice.

Further reinforcing this potential, the reinjection study revealed that 50% of dogs exhibited no OA progression on imaging over 12–18 months post-second injection, with stable osteophyte size and cartilage thickness contrasting starkly with the expected worsening in untreated OA. This structural preservation underscores HTM’s capacity to affect disease advancement, likely by maintaining suppression of synovial inflammation and macrophage activity following reinjection. Unlike nonsteroidal anti-inflammatory drugs (NSAIDs), which alleviate pain but risk gastrointestinal and renal toxicity over time, or corticosteroids which offer short-term relief while accelerating cartilage damage with repeated use (22), Sn-117m delivers targeted joint inflamed inflammatory mediator reduction with minimal systemic effects, leveraging its 0.2–0.3 mm penetration range—ideal for canine elbows—versus Y-90’s broader 3–11 mm reach that risks collateral structures harm.

The Grade 1and 2 study’s durability implies a “therapeutic reset” of the joint’s inflammatory setpoint, reducing macrophage-driven cytokine production and mitigating the inflammatory cycle that drives OA. This hypothesis could be tested with longitudinal histological analysis—examining synovial cellularity and cartilage health—or biomarker assays tracking cartilage oligomeric matrix protein levels, which reflect cartilage turnover. Such data would further elucidate whether Sn-117m’s effects extend to long-term chondroprotection, a consideration for DMOADevice classification.

Radiosynoviorthesis can be effectively integrated into a comprehensive canine osteoarthritis management protocol as a local and targeted therapeutic intervention while minimizing systemic exposure. The treatment integrates seamlessly with concurrent therapies including weight management, controlled exercise protocols, physical rehabilitation, systemic analgesics, or regenerative modalities. Analgesics can provide symptomatic relief as needed, regenerative therapies may potentially encourage angiogenesis and chondrocyte proliferation, while RSO can address progressive degeneration by reducing production of inflammatory mediators and destructive enzymes. Optimal timing for RSO is typically before severe cartilage destruction occurs, making it particularly valuable for maintaining joint structure in mild to moderate osteoarthritis. Even in advanced OA, RSO may help provide sustained symptom relief and slow further progression, while possibly reducing reliance on long-term systemic analgesics.

Challenges persist, however. Optimal dosing varies by joint size and OA severity, as evidenced by the Grade 3 study’s less robust response—73% improvement in pain severity scores at 12 months post treatment as seen by the patient daily caretakers versus 85% in Grade 1and 2 patients showing improvement in pain severity scoring at 12 months post treatment again by the patients daily caretakers—suggesting effectiveness wanes with advanced structural damage or direct stimulation of nociceptors in underlying subchondral bone after cartilage erosion. Early intervention is favored for maximal short and long-term benefits.

Sn-117m’s dual action—offering symptomatic relief and potential structural preservation—positions it to redefine OA management in companion animals. By targeting synovitis, the disease’s inflammatory engine, it addresses a critical therapeutic gap, offering a model for human OA translation and a pathway to improved quality of life for veterinary patients.

## Conclusion

Sn-117m RSO is an emerging therapy for OA, targeting synovitis to disrupt the inflammatory cycle and modify disease progression across mammalian species. Preclinical studies using male Lewis rats with meniscal tear-induced OA demonstrated histopathological improvements—reduced synovial inflammation, cartilage damage, and osteophytes with no deleterious effects --establishing HTM’s effectiveness in a controlled setting. These findings gain clinical weight through canine elbow OA trials: the Grade 1and 2, and the Grade 3 elbow OA studies revealed durable lameness improvement over 12 months, persisting long after Sn-117m’s 13.9-day t_½_, suggesting structural modification beyond transient radiation effects. The reinjection study further bolsters this, with 50% of dogs showing no OA progression on imaging over 12–18 months, contrasting sharply with untreated OA’s typical structural decline.

By directly addressing OA’s inflammatory etiology, Sn-117m may augment or replace symptom-focused treatments, positioning itself as a DMOADevice candidate for veterinary medicine. Its mechanism—low-energy electrons eliminating synovial inflammatory cells reduces macrophage-driven inflammation and preserves joint integrity, delivering both functional relief and structural stability. These dual benefits highlight Sn-117m’s ability to enhance quality of life in companion animals, filling a long-standing gap in effective OA management.

Further investigation is crucial to refine dosing strategies, confirm long-term safety, and benchmark effectiveness against existing modalities. Leveraging human RSO precedents will be essential for widespread veterinary adoption. If substantiated, Sn-117m could transform OA care, with implications extending to human medicine. This review underscores its promise as a pioneering intervention, uniting preclinical rigor with clinical impact to redefine therapeutic possibilities for disease modification.
